# Urinary active transforming growth factor β in feline chronic kidney disease

**DOI:** 10.1016/j.tvjl.2016.02.004

**Published:** 2016-08

**Authors:** J.S. Lawson, H.M. Syme, C.P.D. Wheeler-Jones, J. Elliott

**Affiliations:** a Comparative Biomedical Sciences, The Royal Veterinary College, Royal College Street, London, NW1 0TU, UK; b Clinical Sciences and Services, The Royal Veterinary College, Hawkshead Lane, North Mymms, Hatfield, Herts, AL9 7TA, UK

**Keywords:** Renal, Urine, Cytokine, Fibrosis, Cat

## Abstract

The cytokine transforming growth factor beta 1 (TGF-β1) has been widely implicated in the development and progression of renal fibrosis in chronic kidney disease (CKD) in humans and in experimental models. The aims of this study were to assess the association between urinary active TGF-β1 and (a) development of CKD in a cross-sectional study, (b) deterioration of renal function over 1 year in a longitudinal study, and (c) renal histopathological parameters in cats. A human active TGF-β1 ELISA was validated for use in feline urine.

Cross-sectional analysis revealed no significant difference in urinary active TGF-β1:creatinine ratio (aTGF-β1:UCr) between groups with differing renal function. Longitudinally, non-azotaemic cats that developed CKD demonstrated a significant (*P* = 0.028) increase in aTGF-β1:UCr approximately 6 months before the development of azotaemia, which remained elevated (*P* = 0.046) at diagnosis (approximately 12 months prior, 8.4 pg/mg; approximately 6 months prior, 22.2 pg/mg; at CKD diagnosis, 24.6 pg/mg). In the histopathology study, aTGF-β1:UCr was significantly higher in cats with moderate (*P* = 0.02) and diffuse (*P* = 0.005) renal fibrosis than in cats without fibrosis. Cats with moderate renal inflammation had significantly higher urinary active aTGF-β1 concentrations than cats with mild (*P* = 0.035) or no inflammatory change (*P* = 0.004). The parameter aTGF-β1:UCr was independently associated with Log urine protein:creatinine ratio in a multivariable analysis of clinicopathological parameters and interstitial fibrosis score in a multivariable analysis of histopathological features. These results suggest that urinary aTGF-β1 reflects the severity of renal pathology. Increases in urinary aTGF-β1 followed longitudinally in individual cats may indicate the development of CKD.

## Highlights

• Feline urinary active transforming growth factor beta 1 (aTGF-β1) was measured in healthy and azotaemic cats.
• There was no cross-sectional association between aTGF-β1 and development of chronic kidney disease.
• Elevations in aTGF-β1 preceded development of azotaemia in a longitudinal study.
• Urinary aTGF-β1 concentrations reflected severity of renal pathology.

## Introduction

Chronic kidney disease (CKD) is common in ageing cats, with a prevalence of 28–50% (Lulich et al, 1992, Marino et al, 2014). In most cases, the inciting cause is unknown and the predominant morphological diagnosis is chronic tubulointerstitial inflammation and fibrosis (DiBartola et al, 1987, Chakrabarti et al, 2013).

The cytokine transforming growth factor beta one (TGF-β1) is the most studied pro-fibrotic mediator in human and experimentally-induced kidney disease. TGF-β1 is secreted as an inactive precursor combined with the latency associated pro-peptide and latent TGF-β binding protein as the large latent complex (LLC). The active TGF-β1 peptide must be liberated from the LLC to exert a biological effect, a process that is initiated by a variety of molecules implicated in extra-cellular matrix (ECM) disruption (Annes et al., 2003). The TGF-β1 pathway integrates the effects of many other fibrogenic factors (Liu, 2006) and is upregulated by various renal insults. The pro-fibrotic effects of TGF-β1 include induction of myofibroblast formation (Serini et al, 1998, He et al, 2013, Wu et al, 2013), direct stimulation of ECM-related gene transcription (Border and Noble, 1993) and reduced ECM degradation (Edwards et al, 1987, Krag et al, 2005). Urinary total TGF-β1 excretion increases in various human renal diseases (De Muro et al., 2004) as a result of increased renal production (Sharma et al., 1997).

Previous studies have demonstrated that total TGF-β1 (normalised to urinary creatinine) is also present in increased concentrations in the urine of cats with CKD (Arata et al, 2005, Habenicht et al, 2013), suggesting urinary TGF-β1 may be a useful biomarker of renal injury in feline CKD. However, there are no published studies examining urinary TGF-β1 prior to the diagnosis of azotaemic feline CKD or attempts to correlate urinary TGF-β1 with renal histopathology.

In this study, we hypothesised that urinary active TGF-β1 could be used as a biomarker of renal fibrosis and progressive renal disease in cats. To address this hypothesis, an assay for urinary active TGF-β1 was validated and the relationships between azotaemic CKD and renal fibrosis were assessed. Three analyses were undertaken: a cross-sectional study; a longitudinal study; and a study correlating urinary TGF-β1 with renal histopathology.

## Materials and methods

### Case selection

The records from two first-opinion practices were searched for cats >9 years old with and without renal azotaemia. Renal azotaemia was defined as a plasma creatinine concentration of >177 µmol/L with concurrent urine specific gravity of <1.035. Cats with hyperthyroidism (plasma TT4 >40 nmol/L), urinary tract infection, or other systemic disease diagnosed within the follow-up period were excluded.

For the cross-sectional study, cases were classified based upon baseline status and subsequent renal function, resulting in three groups: non-azotaemic cats that remained non-azotaemic for 24 months (NA), non-azotaemic cats that developed azotaemia within 24 months (NA-A), and cats with renal azotaemia at baseline (A).

For the longitudinal study, cats with three urine samples approximately 6 months apart were identified and classified into three groups: non-azotaemic cats that remained non-azotaemic (stable group), non-azotaemic cats that developed azotaemia after 11–15 months (developed azotaemia group) and azotaemic cats with progression of CKD after 11–15 months (progressive CKD group). Progression was defined as a repeatable >25% increase in plasma creatinine from baseline.

The renal pathology study included cats of any age with and without CKD if histopathological data were available, and a urine sample was collected within 3 months before death. Renal tissue was fixed in 10% neutral buffered formalin and histopathological data were available from a previous study where transverse sections were scored for a variety of pathological features (Chakrabarti et al., 2013). The sections were scored for interstitial fibrosis and inflammation as follows: 0, none or rare small foci; 1, mild or scattered multifocal areas affecting <5% of the section; 2, moderate fibrosis/inflammation affecting 25–50% of the section; 3, diffuse or coalescing fibrosis/inflammation. Glomerulosclerosis was scored as follows: 0, normal (matrix could encircle no more than 1 nucleus); 1, mild (matrix could surround several nuclei but not extend to peripheral capillary loops); 2, moderate (matrix expansion involving peripheral capillary loops and affecting <50% of the glomerulus); and 3, severe (matrix expansion affecting >50% of the glomerulus). Mean glomerular volume was calculated from the cross-sectional area of 20 randomly selected non-obsolescent outer cortical glomerular tufts.

### Clinicopathological data

Blood and urine samples were obtained by jugular venepuncture and cystocentesis, respectively. Urine sediment examination was performed in-house. Samples with evidence of urinary tract infection (bacturia/pyuria or positive urine culture, which was routinely performed when infection was suspected) were excluded. Plasma biochemistry, urine creatinine and urine protein were measured at an external laboratory (IDEXX Laboratories). Residual samples of urine and plasma were retained for research use with owner informed consent. When animals were euthanased and a post-mortem examination was undertaken, owner informed consent was obtained. The project protocol, owner information document and informed consent forms were approved by the RVC's Ethics and Welfare Committee (URN 2013 1258, 2 December 2013).

All urine samples were centrifuged and stored at −80 °C. TGF-β1 concentrations were normalised as a urine TGF-β1:urine creatinine ratio (TGF-β1:UCr). Systolic blood pressure (SBP) was measured by the Doppler method as per ACVIM consensus guidelines (Brown et al., 2007) and mean SBP calculated from five readings.

### TGF-β1 assay validation

Four enzyme-linked immunoassay (ELISA) kits underwent validation for the measurement of TGF-β1 in feline urine: Multispecies total TGF-β1 (Invitrogen), Human Free Active TGF-β1 (BioLegend), Mouse Latent TGF-β (BioLegend) and Human Latent TGF-β (BioLegend). Precision and reproducibility were determined for samples with a range of TGF-β1 concentrations by calculating inter- and intra-assay coefficients of variation (CV) where assays detected measurable feline TGF-β1. Specificity was assessed by evaluating three serial dilutions of samples along the standard curve. The spike-and-recovery of the Free Active TGF-β1 assay was assessed via spiking urine samples with recombinant active TGF-β1 (Part #78251, BioLegend) at high (250 pg/mL), medium (125 pg/mL) and low (62.5 pg/mL) concentrations and comparing results to expected values. The lower limit of detection (LoD) was established by assaying multiple wells (*n* = 8) of the lowest (0 pg/mL) standard and taking four standard deviations above the mean value (Armbruster and Pry, 2008).

### Statistical analysis

Statistical analysis was carried out using computer software (IBM SPSS Statistics for Windows, Version 20.0). Data were assessed visually for normality. Urinary active TGF-β1 demonstrated a non-Gaussian distribution. Comparisons between groups in the cross-sectional and renal pathology study were made using the Kruskal–Wallis test, with post-hoc pairwise Mann–Whitney *U* tests where appropriate, and correlations between variables were examined using Spearman's correlation coefficient. Comparisons between visits within groups in the longitudinal study were made using the Friedman test with post-hoc pairwise Wilcoxon sign rank tests where appropriate. Significance was set at *P* < .05. Results are reported as median values (range) or mean ± standard deviation (SD), as appropriate.

Univariable linear regression analyses were performed to identify clinicopathological (creatinine, urea, phosphate, PCV, potassium, urine protein:creatinine ratio [UPC]) and histopathological (interstitial fibrosis score, inflammation score, glomerulosclerosis score, glomerular volume) predictors of urinary TGF-β1 concentration in the cross-sectional and renal pathology studies, respectively. Variables significant at the 20% level were included in two separate backward selection multivariable analyses with final significance set at the 5% level. Variables were log-transformed if necessary to meet the assumptions for multivariable linear regression.

## Results

### Validation of the ELISA

The Multispecies Total TGF-β1, Mouse Latent TGF-β, Human Latent TGF-β ELISA kits did not detect TGF-β in feline urine. The Free Active TGF-β1 (aTGF-β1) kit detected measurable levels of cytokine in feline urine and underwent further validation. Intra-assay CV for samples measuring 18, 20, 28 and 62 pg/mL aTGF-β1 (*n* = 4) was 2.3–7.5%. Inter-assay CV for samples measuring 26, 62, 118 and 183 pg/mL aTGF-β1 (*n* = 4) was 4.7–20.9%. Dilutional linearity indicated a mean recovery of 91.6% ± 17.5% (*n* = 4). Spiking urine samples with exogenous recombinant aTGF-β1 produced mean recovery values of 110.9 ± 15.9% (*n* = 3), 118.5 ± 11.3% (*n* = 3) and 117.1 ± 9.5% (*n* = 3) for the low, medium and high spikes, respectively. These recovery values were slightly greater than when spiking sample diluent, suggesting a minor matrix effect of feline urine. The lower LoD was 2.9 pg/mL; therefore, samples assayed with an active TGF-β1 < 2.9 pg/mL were assigned the arbitrary value 1.5 pg/mL (US EPA, 2000).

### Urinary active TGF-β1 cross-sectional study

**Fig. 1:**
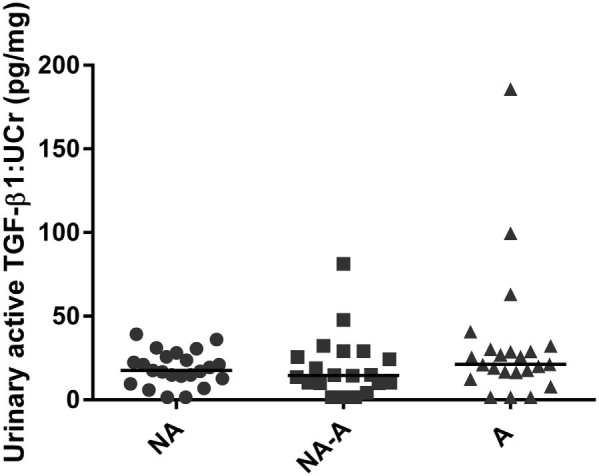
Scatterplot illustrating urinary active transforming growth factor beta 1:urinary creatinine ratio (TGF-β1:UCr) in non-azotaemic cats that remained non-azotaemic for 24 months (NA), non-azotaemic cats that developed azotaemia within 24 months (NA-A) and cats that were azotaemic at baseline (A). The line represents the median. The Kruskal–Wallis test found no significant difference in TGF-β1:UCr between groups (*P* = 0.35).

Sixty-seven cats were included in the cross sectional study. These included 33 male neutered (MN) and 34 female neutered (FN) cats of mean age 13.5 (±2.4) years. There was no difference in aTGF-β1:UCr between groups with differing renal function (NA [*n* = 20], 17.6 pg/mg [1.5–39.0]; NA-A [*n* = 23], 14.5 pg/mg [1.5–81.2]; A [*n* = 24], 21.2 pg/mg [1.5–185.0]; Fig. 1). However, one cat in the NA-A group and three cats in the A group demonstrated an aTGF-β1:UCr at least 50% greater than the highest measured value in the NA group. Considering the whole study population, the only variable correlated with aTGF-β1:UCr was UPC, which showed a weak positive correlation (*r* = 0.257, *P* = 0.036). When separated by group, the only variables significantly (and positively) correlated with TGF-β1:UCr were age (*r* = 0.49, *P* = 0.017), mean SBP (*r* = 0.45, *P* = 0.029) and PCV (*r* = 0.42, *P* = 0.047) in the NA group, and UPC in the A group of cats (*r* = 0.42, *P* = 0.04).

Prior to linear regression, aTGF-β1:UCr, creatinine and UPC were log transformed due to non-Gaussian distribution. Clinicopathological predictors of logaTGF-β1:UCr included in the multivariable model are presented in Table 1. LogUPC was the only independent clinicopathological predictor of logaTGF-β1:UCr (*P* = 0.014).

### Urinary active TGF-β1 longitudinal study

**Fig. 2:**
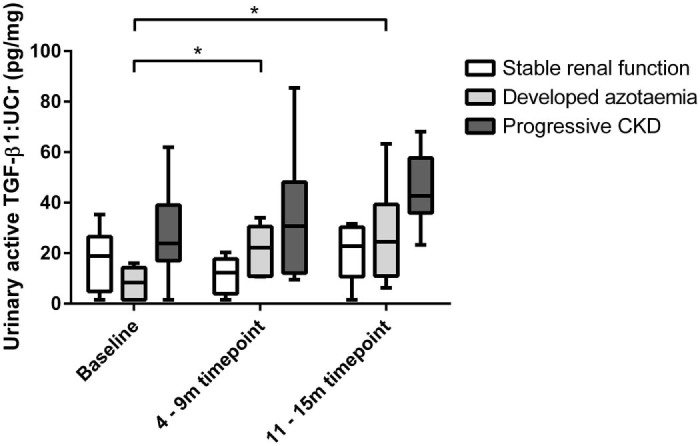
Box and whisker plot illustrating urinary active transforming growth factor beta 1:urinary creatinine ratio (TGF-β1:UCr) in cats with stable renal function over 11–15 months, cats that developed azotaemia at the 11–15 month time-point and cats with progressive CKD at 11–15 months. The boxes represent 25th and 75th percentiles, the central lines represent the median values and the whiskers represent the range. The Friedman test and Wilcoxon sign rank test demonstrated that cats that developed azotaemia demonstrated a significant increase in TGF-β1:UCr approximately 6 months prior to the development of azotaemia (*P* = 0.028), which was sustained at the point of demonstration of azotaemia (*P* = 0.046; *P <* *0.05).

Eighteen cats were included in the longitudinal study, with six cats in each group. These included nine MN and nine FN cats of mean age 13.8 (±2.4) years. Urine samples from three time-points were available – baseline, 4–9 months, and 11–15 months. In the stable renal function group, aTGF-β1:UCr did not change over the course of 1 year (Fig. 2). The developed azotaemia group demonstrated a significant (*P* = 0.028) 2.6-fold increase in aTGF-β1:UCr approximately 6 months prior to development of azotaemia, which remained elevated (*P* = 0.046) at the point of diagnosis of azotaemia. In the progressive CKD group, the aTGF-β1:UCr did not change significantly prior to progression of renal disease.

### Correlation of urinary active TGF-β1 with post-mortem renal pathology

Fifty-nine cats were included in the pathology study (39 MN, one male entire and 19 FN; mean age 13.9 [±5.2] years). Two cats with histopathological diagnoses of papillary necrosis and hydronephrosis, respectively, demonstrated urinary aTGF-β1 concentrations above the highest standard (500 pg/mL) and were assigned a value of 500 pg/mL for the purposes of analysis.

**Fig. 3:**
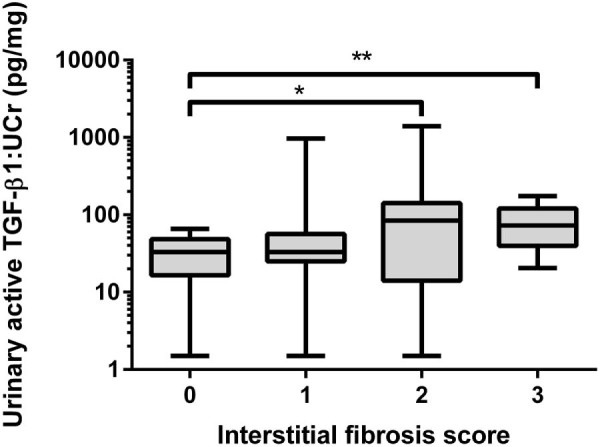
Box and whisker plot illustrating urinary active transforming growth factor beta 1:urinary creatinine ratio (TGF-β1:UCr) of cats in the renal pathology study, grouped by severity of renal interstitial fibrosis. The Kruskal–Wallis test and Mann–Whitney *U* test demonstrated that cats with moderate fibrosis affecting 25–50% of the section (score 2) and diffuse/coalescing fibrosis (score 3) had significantly higher urinary TGF-β1:UCr ratios than cats with no fibrosis.*P <* *0.05; **P <* *0.01.

**Fig. 4:**
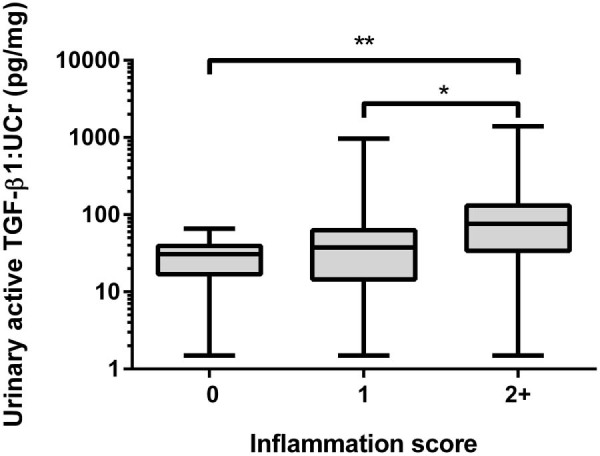
Box and whisker plot illustrating urinary active transforming growth factor beta 1:urinary creatinine ratio (TGF-β1:UCr). The Kruskal–Wallis test and Mann–Whitney *U* test demonstrated that cats with moderate inflammation affecting 25–50% of the section and above (score 2+) had significantly higher urinary TGF-β1:UCr ratios than cats with no inflammatory infiltrate and mild inflammation (score 1). *P <* *0.05; **P <* *0.01.

The parameter aTGF-β1:UCr was moderately correlated with interstitial fibrosis score (*r* = 0.416, *P* = 0.001) and weakly correlated with inflammation score (*r* = 0.385, *P* = 0.003). When grouped by interstitial fibrosis score, cats with scores of 2 (*P* = 0.02) and 3 (*P* = 0.005) had significantly higher aTGF-β1:UCr concentrations than cats that scored 0 for interstitial fibrosis (Fig. 3). When grouped by inflammation score, cats that scored ≥2 had significantly higher aTGF-β1:UCr concentrations than cats that scored 1 (*P* = 0.035) or 0 (*P* = 0.004; Fig. 4). The parameter aTGF-β1:UCr and mean glomerular volume were log transformed prior to linear regression due to non-Gaussian distribution. Histopathological predictors of LogaTGF-β1:UCr included in the multivariable model are presented in Table 2. Fibrosis score was the only independent histopathological predictor of LogaTGF-β1:UCr (*P* = 0.008).

## Discussion

The Free Active TGF-β1 ELISA was the only assay investigated, which detected measurable levels of cytokine, and to our knowledge, this study is the first to measure concentrations of aTGF-β1 in feline urine. The assay demonstrated acceptable precision and reproducibility, specificity and spike-recovery, supporting its use in feline urine, although a western blot would be required to confirm cross-reactivity. The Multispecies Total TGF-β1 assay used previously to measure feline urinary Total TGF-β1 (Arata et al, 2005, Habenicht et al, 2013) did not detect measurable concentrations of feline total TGF-β1 in the present study.

The cross-sectional study failed to detect differences in urinary aTGF-β1 concentration between groups with differing renal function. One cat that became azotaemic and three cats with pre-existing azotaemia demonstrated elevated concentrations of urinary aTGF-β1, but the majority had low concentrations comparable to non-azotaemic cats. There is limited information on urinary aTGF-β1 in the veterinary and medical literature. A previous study in humans detected urinary aTGF-β1 in 25% of patients with active nephropathy subsequent to allograft dysfunction, but aTGF-β1 was undetectable in the urine of patients with normal renal function (Rogier et al., 2005). In contrast, the majority of healthy geriatric cats in this study demonstrated low, but detectable, concentrations of aTGF-β1. This discrepancy could be due to the advanced age of the healthy control group in the current study. Renal TGF-β1 activity is upregulated in aged rodents (Ruiz-Torres et al., 1998) and the same may be true in cats. However, undetected early renal dysfunction in the non-azotaemic group cannot be discounted, despite the 2-year follow-up period.

The findings of this study regarding urinary aTGF-β1 contrast with two previously published feline studies where total TGF-β1 was measured and increased urinary concentrations were present in cats with CKD (Arata et al, 2005, Habenicht et al, 2013). The reason for this discrepancy may be associated with the form of TGF-β1 measured. The concentration of aTGF-β1 in biological fluids is very low compared to total TGF-β1 (Khan et al., 2012) and the two forms may not correlate with each other. While CKD results in increased urinary total TGF-β1 concentrations, as demonstrated previously, the increase may be primarily attributable to increased excretion of the latent complex, rather than the active peptide. Measurement of aTGF-β1 is potentially a more valid estimation of kidney TGF-β1 activity than total TGF-β1, as liberation of aTGF-β1 from the LLC is a pivotal point of control in the regulation of TGF-β1 signalling (Annes et al., 2003). Furthermore, aTGF-β1 has a short plasma half-life, undergoing rapid hepatic clearance (Wakefield et al., 1990), and thus urinary aTGF-β1 is likely to originate locally.

CKD in cats might not induce increased TGF-β1 activity within renal tissue. This would be unexpected, as alterations within the renal micro-environment that occur within the diseased feline kidney, such as increased single nephron filtration rate (Brown and Brown, 1995), oxidative stress (Keegan and Webb, 2010) and RAAS activation (Watanabe and Mishina, 2007), upregulate TGF-β1 production and activation in other species (Wolf, 2006, Shin et al, 2008, Rohatgi, Flores, 2010) . Furthermore, this assumption was not supported by the longitudinal study. Non-azotaemic cats that developed CKD demonstrated a sustained increase in urinary aTGF-β1 excretion approximately 6 months prior to the deterioration of renal function, whereas excretion of aTGF-β1 in non-azotaemic cats with stable renal function and azotaemic cats with progressive CKD did not change over 12 months. Although there was a significant overlap between groups, suggesting a high level of individual variation in aTGF-β1 excretion, this finding suggests that elevations in urinary aTGF-β1 might only be identified during certain phases of CKD pathogenesis, rather than being consistently present in all cats with CKD.

LogUPC was the only independent clinicopathological predictor of urinary aTGF-β1 concentration. In addition, the four cats in the cross-sectional study with elevated aTGF-β1 were either proteinuric or borderline proteinuric. This is in accordance with the medical literature, where urinary TGF-β1 excretion correlated with degree of proteinuria in various glomerulonephropathies (Ellis et al, 1998, Shaker, Sadik, 2013). UPC in cats is an independent predictor of CKD progression (Chakrabarti et al., 2012) and survival (Syme et al., 2006), and if increased urine protein is a contributing factor to, rather than a marker of, progressive renal damage, some of the negative effects could be mediated by the upregulation of inflammatory or pro-fibrotic mediators, such as TGF-β1. Alternatively, increased urinary concentration of TGF-β1 in proteinuric cats may reflect increased fractional excretion of serum TGF-β1 due to the compromised filtration barrier in these cats.

To the authors' knowledge, this is the first study to correlate urinary TGF-β1 with renal histopathology in cats. Cats with higher interstitial fibrosis and inflammation scores had significantly higher urinary aTGF-β1 concentrations than cats where no inflammation or fibrosis was present. Interstitial fibrosis was the only histopathological variable independently associated with urinary aTGF-β1. This agrees with previous studies in humans with glomerular diseases, where urinary TGF-β1 reflected grade of interstitial fibrosis andinflammation (Honkanen et al, 1997, Murakami et al, 1997). Overall, evidence from human studies suggests that severe urinary total TGF-β1 elevations are associated with acute phases of tissue damage and inflammatory glomerular conditions rather than primarily interstitial or slowly progressive diseases (De Muro et al, 2004, Dukanovic et al, 2009). When renal biopsies are submitted for histopathological examination from cats with CKD, a histopathological diagnosis of tubulointerstitial fibrosis is usually made (Chakrabarti et al., 2013), and a substantial number of cats with CKD have slowly progressive or non-progressive disease (Chakrabarti et al., 2012). Additionally, where progression does take place, it may occur in a stepwise rather than a linear fashion (Elliott et al., 2003). These factors might explain why the majority of cats in our renal pathology and cross-sectional studies did not demonstrate substantial elevations of urinary aTGF-β1. The two cats in the renal pathology study with extreme elevations of urinary aTGF-β1 had evidence of acute renal insult, which further supports this hypothesis.

A limitation of the renal pathology study was the varying time (0–90 days) between urine sample collection and post-mortem examination. Acute changes in urinary aTGF-β1 prior to death could have been missed because of this variation. A limitation of all three studies was the use of creatinine as an indicator of renal function. Serum creatinine is an insensitive marker of mild to moderate reductions in glomerular filtration rate (Swan, 1997), and pathological changes in the kidney can be advanced at the time of CKD diagnosis when serum creatinine is used as the primary diagnostic criterion. Additionally, to what extent urinary aTGF-β1 concentration represents the renal activity of this cytokine remains unknown.

## Conclusions

No significant difference was found between cats with differing degrees of renal function in the cross-sectional study, but urinary aTGF-β1 increased prior to documented deterioration of renal function in healthy cats that developed CKD in the longitudinal analysis. Additionally, urinary aTGF-β1 correlated with histopathological evidence of renal inflammation and interstitial fibrosis. These results suggest that urinary aTGF-β1 reflects the severity of renal pathology and that urinary aTGF-β1 followed longitudinally in an individual cat may be a surrogate marker of active renal fibrosis and chronic inflammation that could lead to the development of CKD.

## Conflict of interest statement

JSL is in receipt of a BBSRC CASE studentship co-funded by Elanco Ltd. but they played no role in the study design, in data collection, analysis and interpretation, or in the manuscript writing or submission for publication. JE is a member of the International Renal Interest Society, which is sponsored by Elanco Ltd. None of the authors has any other financial or personal relationships that could inappropriately influence or bias the content of the paper.

## Figures and Tables

**Table 1:** Univariable and multivariable linear regression to identify predictors of urinary active transforming growth factor beta 1:urinary creatinine ratio (TGF-β1:UCr) in the cross-sectional study. Parameters significant at the <0.2 level are presented below. LogUPC was the only independent predictor in the multivariable analysis (*P* = 0.014).

Variable	Univariable analysis				Multivariable analysis			
B	SE	*P*	95% CI for B	B	SE	*P*	95% CI for B
LogUPC	0.553	0.218	0.014	0.117–0.989	0.553	0.218	0.014	0.117–0.989
SBP	0.004	0.002	0.084	−0.001 to 0.009				
Age	0.055	0.025	0.03	0.006–0.105				

B, coefficient; SE, standard error; CI, confidence interval.

**Table 2:** Univariable and multivariable linear regression to identify independent histopathological predictors of urinary active transforming growth factor beta 1:urinary creatinine ratio (TGF-β1:UCr) in the renal pathology study. Parameters significant at the <0.2 level are presented below. Interstitial fibrosis score was the only independent predictor in the multivariable analysis (*P* = 0.008).

Variable	Univariable analysis				Multivariable analysis			
B	SE	*P*	95% CI for B	B	SE	*P*	95% CI for B
Interstitial fibrosis	0.199	0.073	0.008	0.054–0.345	0.199	0.073	0.008	0.054–0.345
Interstitial inflammation	0.243	0.098	0.016	0.046–0.441				
Glomerulosclerosis	0.278	0.168	0.103	−0.058 to 0.614				

B, coefficient; SE, standard error; CI, confidence interval.
